# Supplementary material for: Long-term monitoring of two endangered freshwater mussels (Bivalvia: Unionidae) reveals how demographic vital rates are influenced by species life history traits
Source: PLoS One. 2021 Aug 27;16(8):e0256279. doi: 10.1371/journal.pone.0256279 (PMC8396791; doi:10.1371/journal.pone.0256279)
Supplement: S1 File — (PDF) [file pone.0256279.s001.pdf]

Supporting Information Files (S1-11 Files)

(A) ***Epioblasma brevidens***

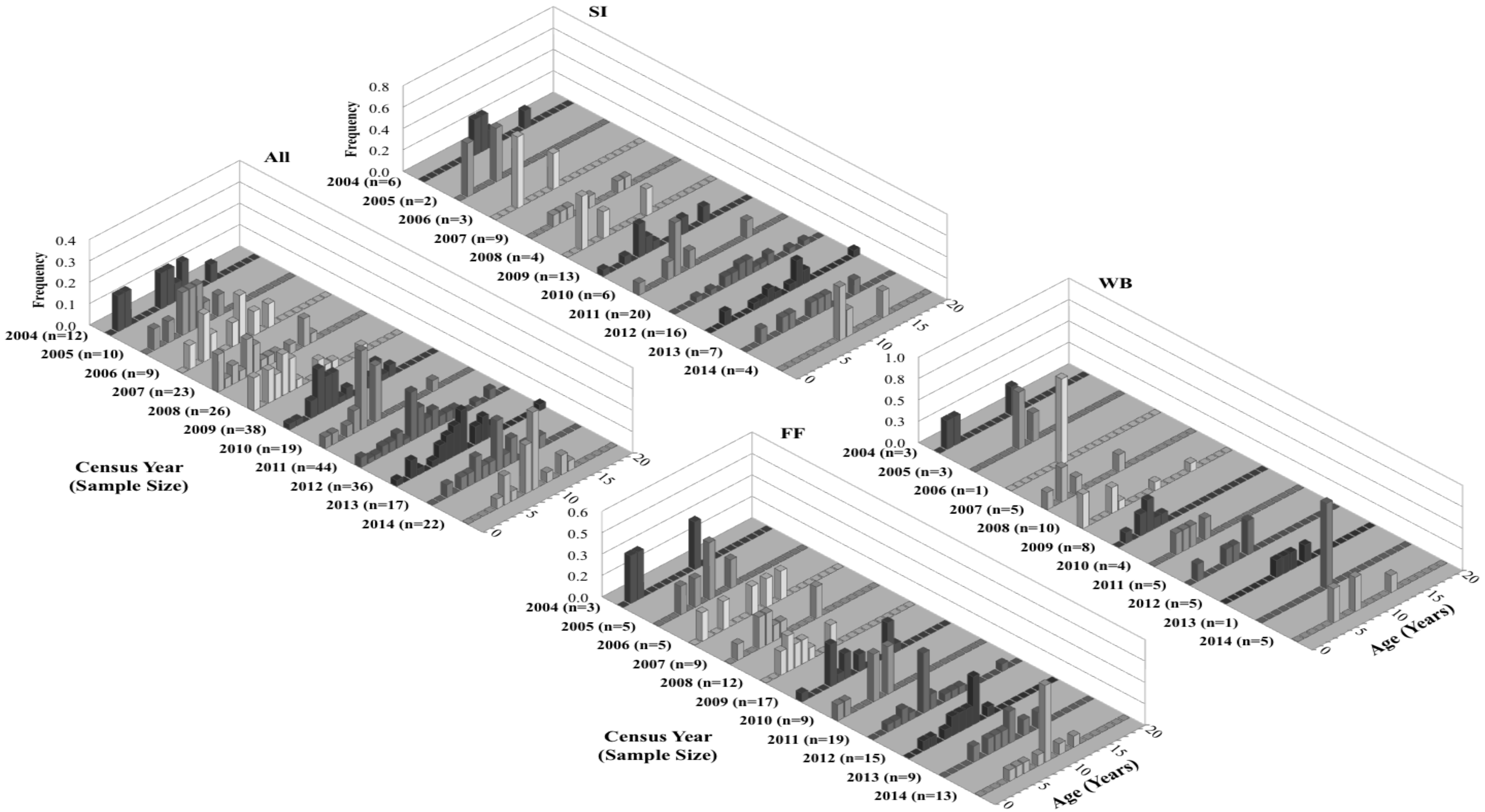

(B) ***Epioblasma capsaeformis***

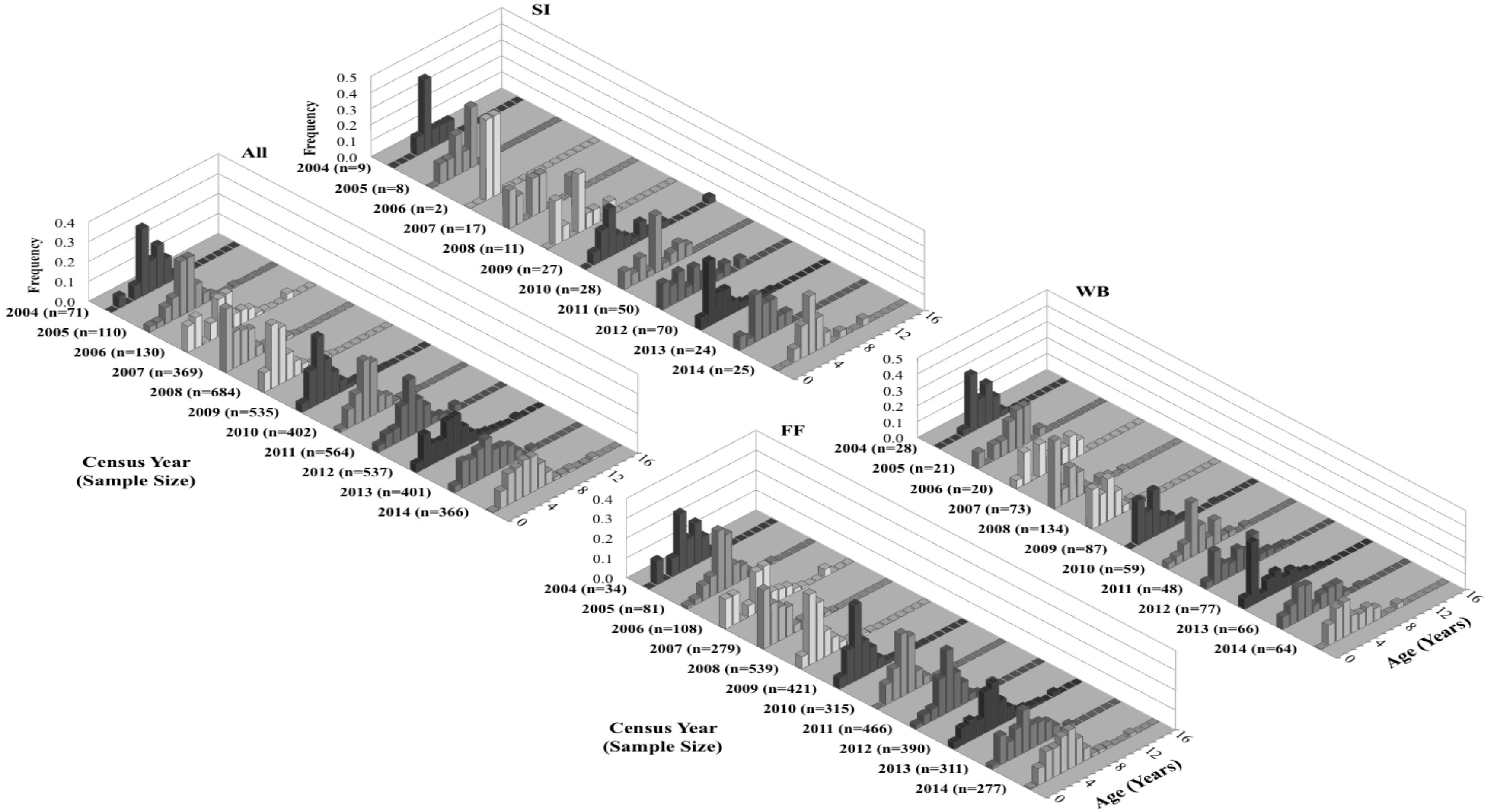

S1. Relative frequencies of age classes in samples of (**A**) *Epioblasma brevidens* and (**B**) *Epioblasma capsaeformis* at Frost Ford (FF), Swan Island (SI), Wallen Bend (WB), and individuals added together across all three sites (All) in the Clinch River, Hancock County, TN, from 2004–2014. Ages were predicted from shell length (mm) using sex-specific von Bertalanffy growth-curve equations in Jones and Neves (2011).
