# Supplementary material for: Long-term monitoring of two endangered freshwater mussels (Bivalvia: Unionidae) reveals how demographic vital rates are influenced by species life history traits
Source: PLoS One. 2021 Aug 27;16(8):e0256279. doi: 10.1371/journal.pone.0256279 (PMC8396791; doi:10.1371/journal.pone.0256279)
Supplement: S2 File — (PDF) [file pone.0256279.s002.pdf]

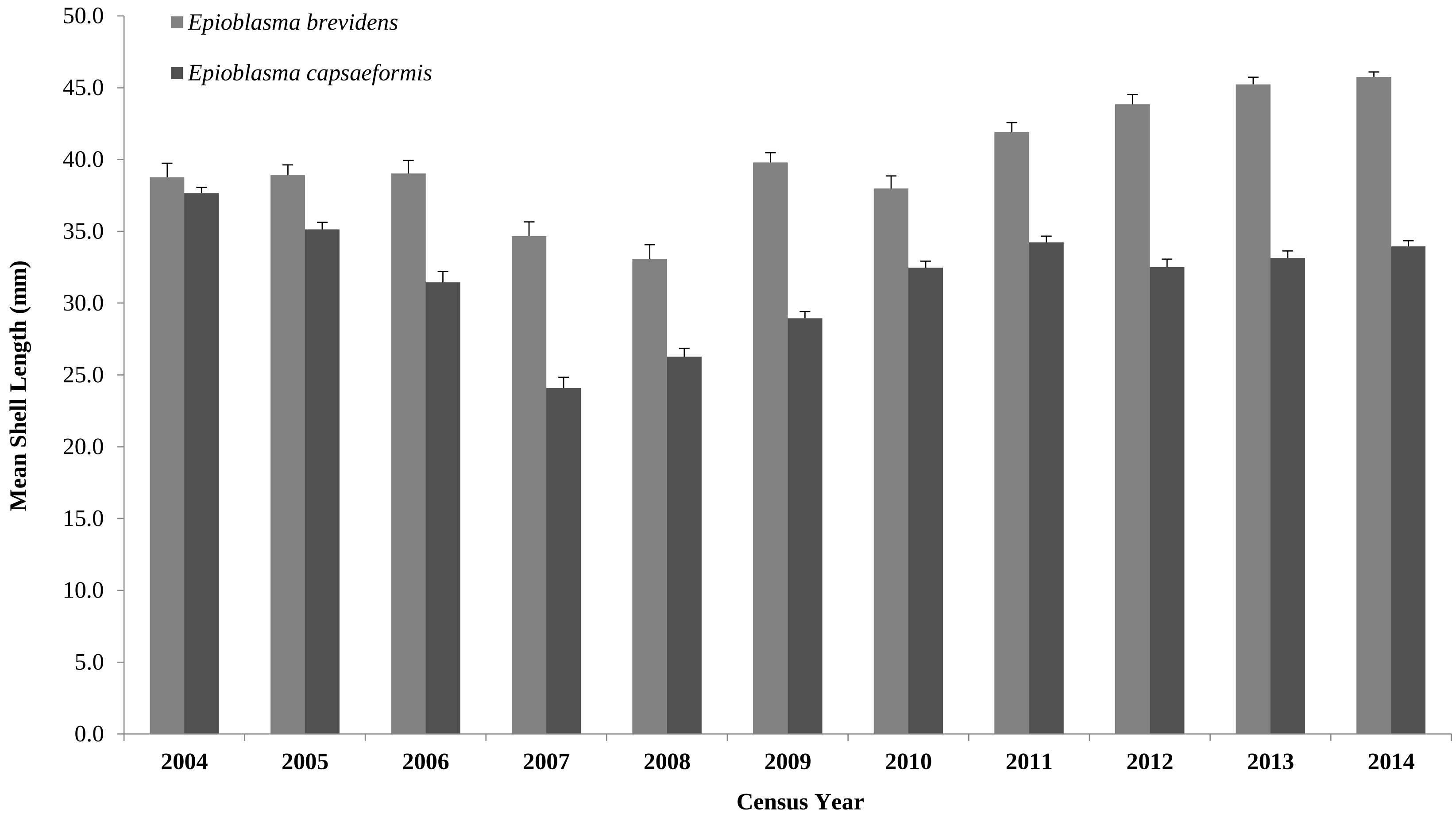

S2 File. Mean lengths (mm) of all live *Epioblasma brevidens* and *E. capsaeformis* in quadrat samples at Frost Ford, Swan Island, and Wallen Bend in the Clinch River, Hancock County, Tennessee from 2004–2014. Error bars are the SE of samples and sample sizes (*N*) are available in Table 2.
