# Supplementary material for: Long-term monitoring of two endangered freshwater mussels (Bivalvia: Unionidae) reveals how demographic vital rates are influenced by species life history traits
Source: PLoS One. 2021 Aug 27;16(8):e0256279. doi: 10.1371/journal.pone.0256279 (PMC8396791; doi:10.1371/journal.pone.0256279)
Supplement: S3 File — (PDF) [file pone.0256279.s003.pdf]

S3 File. Estimated variances for *Epioblasma brevidens* (*Eb*) and *E. capsaeformis* (*Ec*) population abundance ($\hat{N}$) in the Clinch River, TN over time series 2004–2008, 2009–2014, and 2004–2014.include: 1) total variances S^2 ($\hat{N}$), 2) sampling error variances Var ($\hat{N} \mid \mathfrak{F}$), 3) true temporal process variances $\widehat{Var}$ (N), 4) percent sampling variances of total variances %Var ($\hat{N} \mid \mathfrak{F}$), 5) total ($S$) and temporal ($T$) coefficients of variation C_v, and 6) total and temporal standard errors (SE).

	Swan Island			Frost Ford			Wallen Bend			All Sites (Pooled)		
	2004-08	2009-14	2004-14	2004-08	2009-14	2004-14	2004-08	2009-14	2004-14	2004-08	2009-14	2004-14
Epioblasma brevidens												
$\hat{N}$	1,843	3,227	2,598	6,995	10,345	8,822	933	827	876	9,771	14,399	12,295
S^2 ($\hat{N}$),	1,135,411	2,597,035	2,274,676	15,679,520	5,899,201	12,281,918	531,007	246,199	338,570	24,405,618	16,728,696	23,966,247
Var ($\hat{N} \mid \mathfrak{F}$)	764,774	1,420,115	1,122,233	7,650,084	9,203,676	8,497,498	214,984	164,446	187,418	4,963,824	6,416,038	5,755,941
$\widehat{Var}$ (N)	370,637	1,176,920	1,152,443	8,029,436	-3,304,475[a]	3,784,420	316,022	81,753	151,152	19,441,794	10,312,657	18,210,306
% Var ($\hat{N} \mid \mathfrak{F}$)	67	55	49	49	156	69	40	67	55	20	38	24
C_v (S)	0.58	0.50	0.58	0.57	0.23	0.40	0.78	0.60	0.67	0.51	0.28	0.40
C_v (T)	0.33	0.34	0.41	0.41	[a]	0.22	0.60	0.35	0.44	0.45	0.22	0.35
SE_{Total}	477	658	477	1,771	992	1,108	326	203	184	2,209	1,670	1,548
$SE_{Temporal}$	272	443	339	1,267	[a]	615	251	117	123	1,972	1,311	1,349
Epioblasma capsaeformis												
$\hat{N}$	3,917	11,120	7,846	247,852	280,100	265,442	11,837	11,593	11,704	263,606	302,813	284,992
S^2 ($\hat{N}$),	5,411,635	24,965,837	28,798,324	49,610,954,542	2,576,918,661	21,416,451,905	110,125,349	15,311,245	51,721,959	54,866,408,988	2,889,078,924	23,810,337,991
Var ($\hat{N} \mid \mathfrak{F}$)	1,611,809	5,471,311	3,716,992	969,178,840	678,397,426	810,570,796	4,485,370	3,919,634	4,176,787	539,968,073	381,159,001	453,344,943
$\widehat{Var}$ (N)	3,799,826	19,494,526	25,081,332	48,641,775,702	1,898,521,235	20,605,881,108	105,639,978	11,391,611	47,545,172	54,326,440,916	2,507,919,923	23,356,993,048
% Var ($\hat{N} \mid \mathfrak{F}$)	30	22	13	2	26	4	4	26	8	1	13	2
C_v (S)	0.59	0.45	0.68	0.90	0.18	0.55	0.89	0.34	0.61	0.89	0.18	0.54
C_v (T)	0.50	0.40	0.64	0.89	0.16	0.54	0.87	0.29	0.59	0.88	0.17	0.54
SE_{Total}	1,040	2,040	1,697	99,610	20,724	46,278	4,693	1,597	2,274	104,753	21,943	48,796
$SE_{Temporal}$	872	1,803	1,584	98,632	17,788	45,394	4,597	1,378	2,180	104,237	20,445	48,329

[a] Inadmissible estimate of temporal variance
