# Supplementary material for: Long-term monitoring of two endangered freshwater mussels (Bivalvia: Unionidae) reveals how demographic vital rates are influenced by species life history traits
Source: PLoS One. 2021 Aug 27;16(8):e0256279. doi: 10.1371/journal.pone.0256279 (PMC8396791; doi:10.1371/journal.pone.0256279)
Supplement: S4 File — (PDF) [file pone.0256279.s004.pdf]

S4 File. Total number of glochidia brooded per gravid female *Epioblasma brevidens* sampled from Kyles Ford, Clinch River, Hancock County, Tennessee in spring 2013. Ages were estimated based on total shell length, using the von Bertalanffy growth-curve equation for females presented in Jones and Neves (2011).

Total length (mm)	Estimated age (years)	No. glochidia
42.1	6	27,814
42.5	6	28,701
43.2	7	37,839
44.0	7	24,960
44.8	7	18,987
45.0	7	33,764
45.1	7	28,390
45.9	8	37,146
46.0	8	33,788
47.1	8	30,108
48.5	9	43,946
49.5	10	31,164
50.0	11	45,853
51.1	12	56,151
52.2	13	45,597
