# Supplementary material for: Long-term monitoring of two endangered freshwater mussels (Bivalvia: Unionidae) reveals how demographic vital rates are influenced by species life history traits
Source: PLoS One. 2021 Aug 27;16(8):e0256279. doi: 10.1371/journal.pone.0256279 (PMC8396791; doi:10.1371/journal.pone.0256279)
Supplement: S5 File — (PDF) [file pone.0256279.s005.pdf]

S5 File. Total number of glochidia brooded per gravid female *Epioblasma capsaeformis* sampled from Kyles Ford, Clinch River, Hancock County, Tennessee in spring 2013. Ages were estimated based on total shell length, using von Bertalanffy growth curve equations for females presented in Jones and Neves (2011). The 2002 data (*) are from Jones (2004).

Year sampled	Total length (mm)	Estimated age (years)	No. glochidia
2002*	36.7	5	10,494
	39.5	5	12,622
	40.0	6	11,750
	40.8	6	14,062
	41.0	6	16,162
	41.5	6	12,374
	42.0	7	7,780
	43.2	7	14,896
	43.5	7	13,068
	46.4	10	16,876
2013	35.0	4	5,788
	35.1	4	5,714
	35.5	4	7,671
	35.5	4	4,715
	37.0	5	9,465
	37.5	5	6,814
	38.5	5	7,577
	38.5	5	9,395
	39.1	5	3,456
	39.5	5	14,310
	40.5	6	6,538
	40.5	6	13,290
	40.5	6	7,471
	41.0	6	10,136
	41.1	6	11,058
	41.5	6	5,918
	42.5	7	10,068
	44.0	8	17,949
	44.5	8	11,636
	46.1	9	22,182
