# Supplementary material for: Long-term monitoring of two endangered freshwater mussels (Bivalvia: Unionidae) reveals how demographic vital rates are influenced by species life history traits
Source: PLoS One. 2021 Aug 27;16(8):e0256279. doi: 10.1371/journal.pone.0256279 (PMC8396791; doi:10.1371/journal.pone.0256279)
Supplement: S6 File — (PDF) [file pone.0256279.s006.pdf]

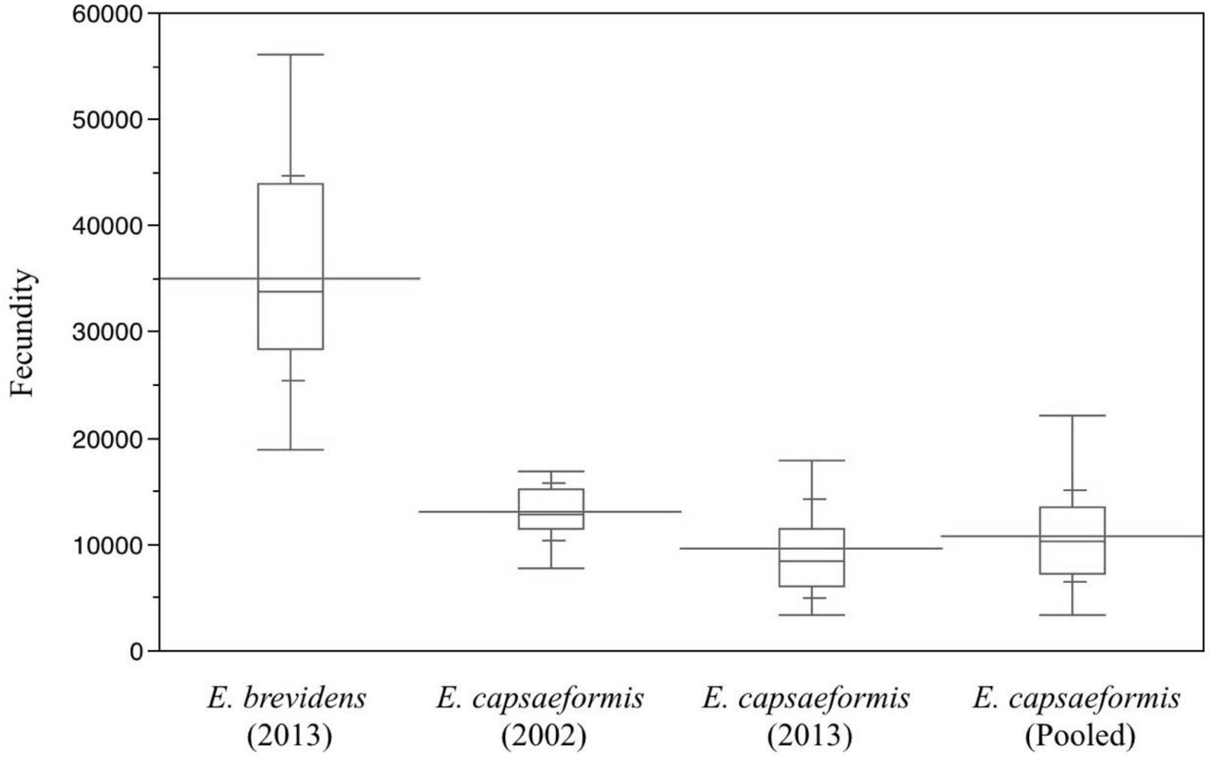

S6 File. Fecundity as number of glochidia in female *Epioblasma brevidens* and *E. capsaeformis* from the Clinch River, Hancock County, Tennessee in 2013 and *E. capsaeformis* from the Clinch River, TN in 2002 (data from Jones, 2004). Box-plots represent quartiles and total range for sample distributions, extended bars indicate sample means, and offset bars indicated one standard deviation.
