# Supplementary material for: Long-term monitoring of two endangered freshwater mussels (Bivalvia: Unionidae) reveals how demographic vital rates are influenced by species life history traits
Source: PLoS One. 2021 Aug 27;16(8):e0256279. doi: 10.1371/journal.pone.0256279 (PMC8396791; doi:10.1371/journal.pone.0256279)
Supplement: S7 File — (PDF) [file pone.0256279.s007.pdf]

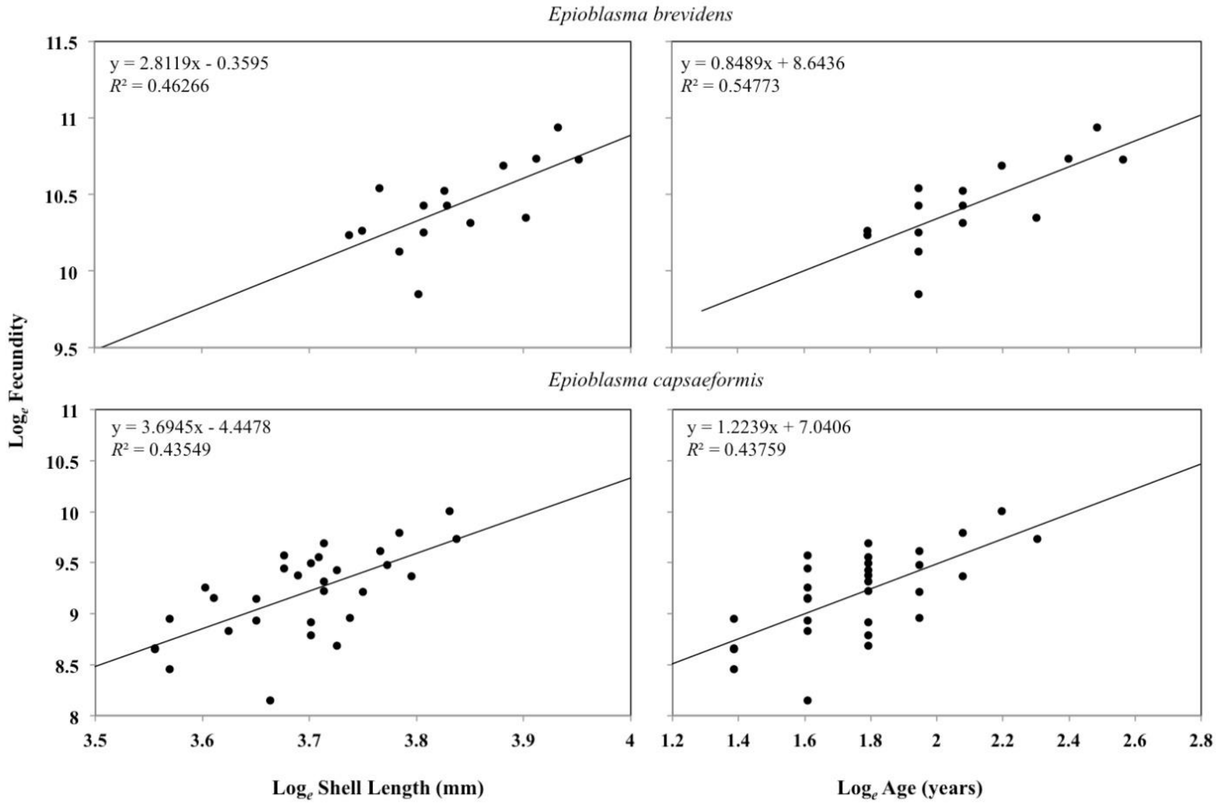

S7 File. Linear relationships between natural logarithm (*Ln*) shell length and *Ln* age and fecundity in *Epioblasma brevidens* and *E. capsaeformis* sampled at Kyles Ford, Clinch River, Hancock County, Tennessee in spring 2013.
