# Supplementary material for: Long-term monitoring of two endangered freshwater mussels (Bivalvia: Unionidae) reveals how demographic vital rates are influenced by species life history traits
Source: PLoS One. 2021 Aug 27;16(8):e0256279. doi: 10.1371/journal.pone.0256279 (PMC8396791; doi:10.1371/journal.pone.0256279)
Supplement: S8 File — (PDF) [file pone.0256279.s008.pdf]

S8 File. Pearson Product-Moment Correlations (Pearson's r) for flow statistics in regular type above with p-values italicized below. Values in bold have a p-value <0.1.

	90DayMin	AprMed	AugMed	Days Extreme High Flow	Days Extreme Low Flow	Days Low Flow + Extreme Low Flows	DaysBelowMed	DecMed	FebMed	JanMed	JulyMed	JuneMed	JuneSeptMed	MarMed	MayMed	MedYear	NovMed	OctMed	SeptMed
90DayMax	**0.873**	0.541	**0.615**	**0.898**	**-0.777**	**-0.783**	**-0.706**	**0.552**	0.537	0.478	**0.672**	0.519	**0.666**	**0.804**	**0.665**	**0.673**	**0.081**	0.143	0.377
	0.001	*0.106*	***0.059***	***0.000***	***0.008***	***0.007***	***0.022***	***0.098***	*0.110*	*0.162*	***0.033***	*0.124*	***0.035***	***0.005***	***0.036***	***0.033***	***0.825***	*0.693*	*0.284*
90DayMin		0.305	**0.875**	**0.786**	**-0.815**	**-0.889**	**-0.873**	**0.615**	**0.699**	**0.708**	**0.841**	**0.703**	**0.843**	**0.593**	**0.561**	**0.862**	0.107	0.189	0.440
		0.392	***0.001***	***0.007***	***0.004***	***0.001***	***0.001***	***0.059***	***0.024***	***0.022***	***0.002***	***0.023***	***0.002***	***0.071***	***0.092***	***0.001***	*0.768*	*0.601*	*0.203*
AprMed			0.125	0.535	-0.325	-0.381	-0.301	-0.130	-0.148	-0.097	0.391	0.338	0.396	**0.673**	**0.811**	0.339	-0.035	-0.136	0.232
			0.730	*0.111*	*0.360*	*0.277*	*0.398*	*0.720*	*0.683*	*0.790*	*0.264*	*0.339*	*0.257*	***0.033***	***0.004***	*0.338*	*0.925*	*0.709*	*0.519*
AugMed				**0.606**	**-0.642**	**-0.694**	**-0.736**	0.495	**0.579**	**0.561**	**0.680**	**0.733**	**0.840**	0.308	0.526	**0.720**	-0.188	-0.081	0.478
				0.063	***0.045***	***0.026***	***0.015***	*0.146*	***0.080***	***0.092***	***0.030***	***0.016***	***0.002***	*0.386*	*0.119*	***0.019***	*0.603*	*0.824*	*0.162*
Days Extreme High Flow					**-0.707**	**-0.803**	**-0.717**	**0.615**	**0.547**	0.421	**0.575**	0.342	0.472	**0.815**	**0.686**	**0.659**	0.176	0.057	0.216
					0.022	***0.005***	***0.020***	***0.058***	***0.100***	*0.226*	***0.082***	*0.333*	*0.169*	***0.004***	***0.028***	***0.038***	*0.626*	*0.875*	*0.549*
Days Extreme Low Flow						**0.741**	**0.876**	**-0.785**	**-0.705**	**-0.827**	**-0.617**	-0.371	**-0.619**	-0.349	-0.464	**-0.777**	-0.291	-0.371	-0.535
						0.014	***0.001***	***0.007***	***0.023***	***0.003***	***0.057***	*0.291*	***0.056***	*0.324*	*0.176*	***0.008***	*0.415*	*0.291*	*0.111*
Days Low Flow + Extreme Low							**0.921**	**-0.642**	**-0.709**	**-0.685**	**-0.812**	**-0.568**	**-0.649**	**-0.644**	**-0.572**	**-0.951**	-0.391	-0.343	-0.140
							0.001	***0.045***	***0.022***	***0.029***	***0.004***	***0.087***	***0.042***	***0.045***	***0.084***	***0.001***	*0.264*	*0.332*	*0.700*
DaysBelowMed								**-0.701**	**-0.683**	**-0.839**	**-0.742**	**-0.560**	**-0.659**	-0.414	-0.525	**-0.969**	-0.446	-0.482	-0.257
								0.024	***0.029***	***0.002***	***0.014***	***0.092***	***0.038***	*0.234*	*0.119*	***0.001***	*0.196*	*0.158*	*0.473*
DecMed									**0.884**	**0.767**	0.332	0.004	0.228	0.176	0.110	**0.589**	0.367	0.352	0.203
									0.001	***0.010***	*0.348*	*0.990*	*0.526*	*0.626*	*0.762*	***0.073***	*0.296*	*0.318*	*0.574*
FebMed										**0.802**	**0.618**	0.117	0.404	0.233	0.017	**0.648**	0.303	0.192	0.373
										0.005	***0.057***	*0.748*	*0.247*	*0.518*	*0.963*	***0.043***	*0.395*	*0.594*	*0.288*
JanMed											**0.633**	0.253	0.460	0.102	0.016	**0.760**	**0.561**	**0.572**	0.364
											0.050	*0.480*	*0.181*	*0.779*	*0.965*	***0.011***	***0.091***	***0.084***	*0.302*
JulyMed												**0.630**	**0.808**	**0.562**	0.414	**0.813**	0.196	0.102	0.512
												0.051	***0.005***	***0.091***	*0.234*	***0.004***	*0.588*	*0.780*	*0.130*
JuneMed													**0.887**	0.334	**0.659**	**0.658**	-0.231	0.086	0.248
													0.001	*0.345*	***0.038***	***0.039***	*0.522*	*0.813*	*0.490*
MedJuneSept														0.376	**0.623**	**0.716**	-0.254	-0.052	**0.619**
														0.284	***0.054***	***0.020***	*0.478*	*0.886*	***0.057***
MarMed															**0.603**	0.440	0.141	-0.032	0.038
															0.065	*0.203*	*0.698*	*0.930*	*0.918*
MayMed																**0.558**	-0.206	-0.101	0.187
																0.093	*0.568*	*0.782*	*0.606*
MedYear																	0.404	0.446	0.209
																	0.247	*0.196*	*0.561*
NovMed																		**0.808**	-0.356
																		0.005	*0.313*
OctMed																			-0.345
																			0.330
