# Supplementary material for: Long-term monitoring of two endangered freshwater mussels (Bivalvia: Unionidae) reveals how demographic vital rates are influenced by species life history traits
Source: PLoS One. 2021 Aug 27;16(8):e0256279. doi: 10.1371/journal.pone.0256279 (PMC8396791; doi:10.1371/journal.pone.0256279)
Supplement: S9 File — (PDF) [file pone.0256279.s009.pdf]

S9 File. Pearson Product-Moment Correlations (Pearson's r) between flow statistics and population parameters for each species, including arcsine square root transformation of portion of juveniles detected in a census, realized population growth calculated using adults only and with juveniles. Pearson' r values are listed in regular type with associated p-values were italicized below. Values in bold have a p-value <0.1.

	90DayMax	90DayMin	AprMed	AugMed	Days Extreme Flow	Days Extreme Low Flow	Days Low Flow + Ex Low Flows	DaysBelowMed	DecMed	FebMed	JanMed	JulyMed
ARCSIN Ebrev	**-0.835**	**-0.882**	-0.261	**-0.694**	**-0.686**	**0.870**	**0.789**	**0.773**	**-0.748**	**-0.792**	**-0.726**	**-0.697**
Prop Juv	***0.003***	***0.001***	*0.466*	***0.026***	***0.029***	***0.001***	***0.007***	***0.009***	***0.013***	***0.006***	***0.018***	***0.025***
ARCSIN Ecaps	**-0.646**	-0.486	**-0.577**	-0.245	**-0.586**	**0.766**	**0.557**	**0.570**	**-0.573**	-0.506	-0.424	-0.435
Prop Juv	***0.044***	*0.154*	***0.081***	*0.495*	***0.075***	***0.010***	***0.094***	***0.085***	***0.084***	*0.136*	*0.222*	*0.209*
Ebrev r(A)	-0.217	-0.410	0.141	-0.486	-0.111	0.171	0.272	0.229	-0.133	-0.395	-0.200	-0.510
	0.547	*0.240*	*0.698*	*0.155*	*0.759*	*0.636*	*0.447*	*0.524*	*0.714*	*0.259*	*0.579*	*0.132*
Ebrev r(A+J	-0.439	**-0.683**	0.007	**-0.745**	-0.421	0.436	**0.630**	**0.612**	-0.390	-0.544	-0.433	**-0.644**
	0.205	***0.029***	*0.985*	***0.013***	*0.225*	*0.208*	***0.051***	***0.060***	*0.265*	*0.104*	*0.211*	***0.045***
Ecaps r(A)	-0.213	-0.449	-0.324	**-0.585**	-0.352	0.245	0.555	0.443	-0.195	-0.410	-0.227	-0.576
	0.554	*0.193*	*0.362*	***0.076***	*0.319*	*0.495*	*0.096*	*0.200*	*0.590*	*0.239*	*0.528*	*0.082*
Ecaps r(A+J)	-0.505	**-0.650**	-0.318	**-0.805**	**-0.659**	0.507	**0.654**	**0.602**	-0.499	-0.514	-0.282	-0.473
	0.136	***0.042***	*0.371*	***0.005***	***0.038***	*0.135*	***0.040***	***0.065***	*0.142*	*0.129*	*0.429*	*0.167*

	JuneMed	JuneSeptMed	MarMed	MayMed	NovMed	OctMed	SeptMed	YearMed	30D Med*	60D Med*	90D Med*
ARCSIN Ebrev	-0.462	**-0.717**	-0.479	-0.421	-0.098	-0.187	-0.491	**-0.741**	**-0.588**	**-0.812**	**-0.811**
Prop Juv	*0.179*	***0.020***	*0.162*	*0.225*	*0.788*	*0.605*	*0.150*	***0.014***	***0.074***	***0.004***	***0.004***
ARCSIN Ecaps	-0.145	-0.407	-0.360	-0.543	-0.152	-0.156	-0.461	-0.547	-0.477	-0.510	-0.531
Prop Juv	*0.690*	*0.243*	*0.306*	*0.105*	*0.676*	*0.666*	*0.180*	*0.101*	*0.163*	*0.132*	*0.114*
Ebrev r(A)	-0.489	-0.547	0.023	-0.123	0.290	0.149	-0.463	-0.332	-0.273	-0.465	-0.520
	0.151	*0.102*	*0.949*	*0.736*	*0.416*	*0.680*	*0.177*	*0.348*	*0.446*	*0.176*	*0.124*
Ebrev r(A+J	**-0.683**	**-0.685**	-0.161	-0.401	0.077	-0.073	-0.314	**-0.690**	-0.176	-0.513	**-0.593**
	0.030	***0.029***	*0.658*	*0.251*	*0.834*	*0.841*	*0.376*	***0.027***	*0.627*	*0.129*	***0.071***
Ecaps r(A)	-0.372	-0.516	-0.271	-0.417	0.120	0.369	-0.242	-0.530	-0.263	-0.476	**-0.562**
	0.290	*0.127*	*0.448*	*0.230*	*0.741*	*0.294*	*0.500*	*0.115*	*0.463*	*0.164*	***0.091***
Ecaps r(A+J)	-0.480	**-0.602**	-0.343	**-0.668**	0.253	0.311	-0.294	**-0.610**	-0.385	**-0.613**	**-0.616**
	0.160	***0.065***	*0.331*	***0.035***	*0.480*	*0.382*	*0.410*	***0.061***	*0.271*	***0.060***	***0.058***

**Post hoc* analysis compared correlations between population parameters and median flow for 30, 60 and 90 days prior to census.
