# Supplementary material for: Long-term monitoring of two endangered freshwater mussels (Bivalvia: Unionidae) reveals how demographic vital rates are influenced by species life history traits
Source: PLoS One. 2021 Aug 27;16(8):e0256279. doi: 10.1371/journal.pone.0256279 (PMC8396791; doi:10.1371/journal.pone.0256279)
Supplement: S10 File — (PDF) [file pone.0256279.s010.pdf]

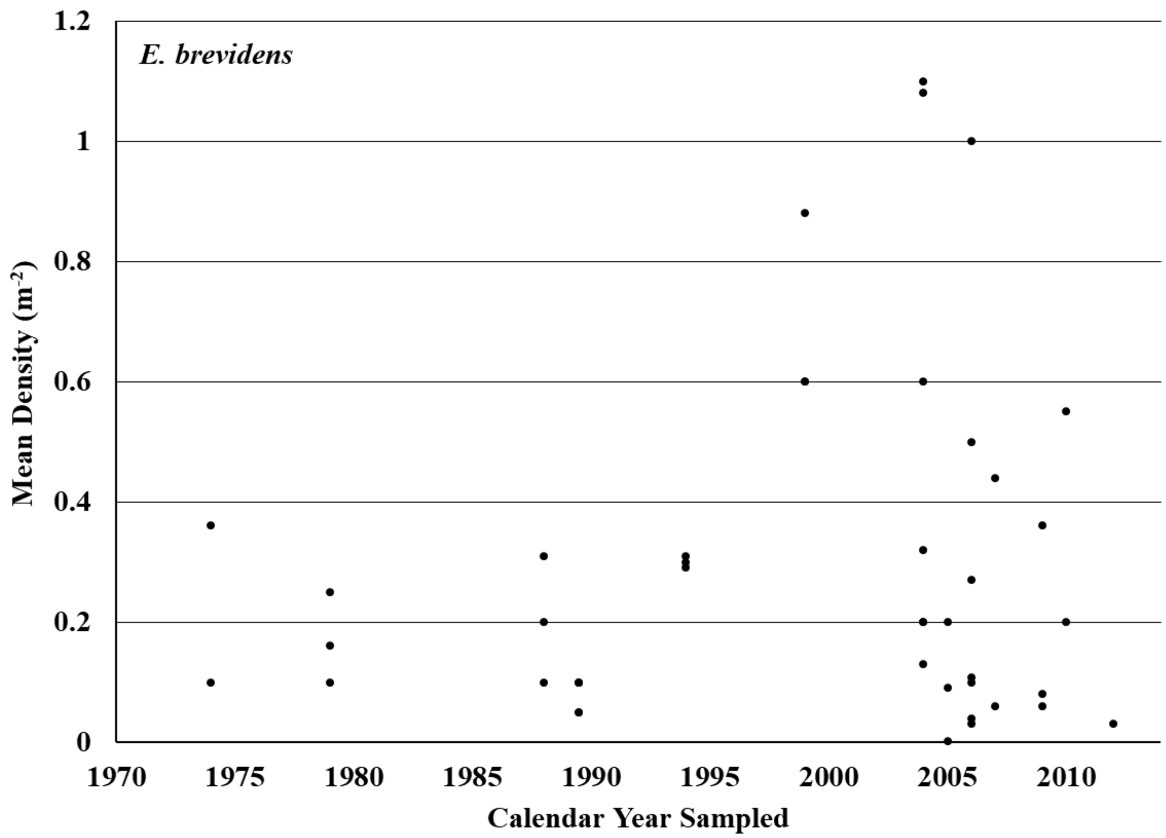
E. brevidens
Mean Density (m-2)
0
0.2
0.4
0.6
0.8
1
1.2
1970
1975
1980
1985
1990
1995
2000
2005
2010
Calendar Year Sampled

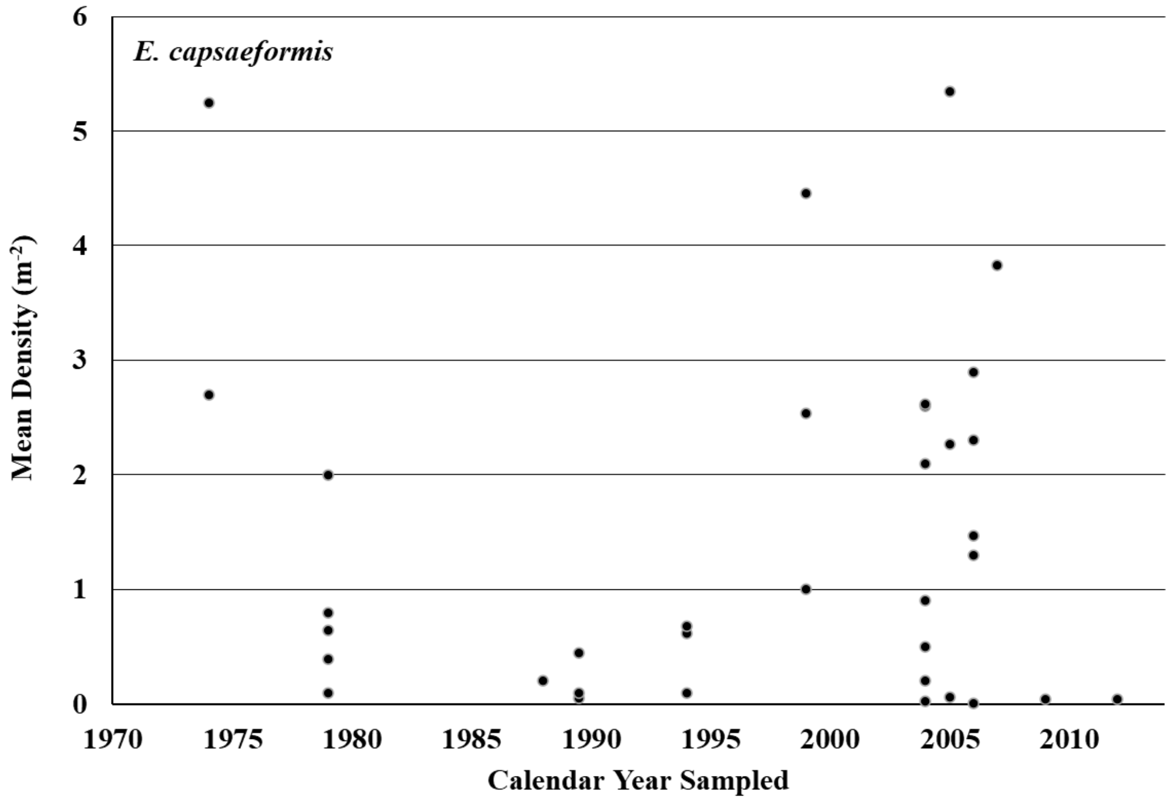
E. capsaeformis
Mean Density (m-2)
0
1
2
3
4
5
6
1970
1975
1980
1985
1990
1995
2000
2005
2010
Calendar Year Sampled

S10 File. Between 1973 and 2014, researchers detected at least one *E. brevidens* during 44 sampling events that sought to quantify mussel density in the Clinch River (top). Mean estimates of density (m^{-2}) for this species as presented in published accounts or determined by analysis of unpublished data are plotted here by calendar year showing that density of *E. brevidens* has rarely exceeded 1 mussel m^{-2}. A similar plot is presented for *E. capsaeformis* showing its density over the same time period. Data sources are presented in Supplementary file S9 below. The S8 figure does not include data from the data presented in the study analysis.
