# Supplementary material for: Long-term monitoring of two endangered freshwater mussels (Bivalvia: Unionidae) reveals how demographic vital rates are influenced by species life history traits
Source: PLoS One. 2021 Aug 27;16(8):e0256279. doi: 10.1371/journal.pone.0256279 (PMC8396791; doi:10.1371/journal.pone.0256279)
Supplement: S11 File — (PDF) [file pone.0256279.s011.pdf]

S11 File. Data sources for mean density (m^{-2}) estimates of *E. brevidens* and *E. capsaeformis* used to construct Figure 15. The river mile (CRM), year data was collected, mean density in m^{-2}, source, and reference number are listed for each point.

CRM	Year	Mean *E. brevidens* Density	Mean *E. capsaeformis* Density	Source	Reference #
172.2	1988	0.2		Ahstedt et al. 2016	1
172.2	1994	0.3	0.1	Ahstedt et al. 2016	1
172.2	1999	0.6	1	Ahstedt et al. 2016	1
172.2	2004	0.6	0.9	Ahstedt et al. 2016	1
174.5	2006	0.1	1.3	Jones et al. 2014	2
178.7	2006	0.5	2.3	Jones et al. 2014	2
179.4	2006	1	2.9	Jones et al. 2014	2
181	2007	0.44	3.83	Jones et al. 2014	2
182.5	2005	0.09	5.35	Jones et al. 2014	2
183.3	1988	0.31		Ahstedt et al. 2016	1
183.3	1994	0.31	0.62	Ahstedt et al. 2016	1
183.3	1999		4.46	Ahstedt et al. 2016	1
183.3	2004	1.1	2.6	Ahstedt et al. 2016	1
183.5	1989-1990	0.1	0.05	Hubbs et al. 1991	37
183.5	2005	0.2	2.27	Jones et al. 2014	2
183.7	2004	1.08	2.62	Jones et al. 2014	2
187.5	2006	0.27	1.47	Jones et al. 2014	2
189.6	1974	0.1	5.25	Dennis 1984	38
189.6	1979	0.1	0.39	Ahstedt et al. 2016	1
189.6	1989-1990	0.05		Hubbs et al. 1991	37
189.6	1994	0.29	0.68	Ahstedt et al. 2016	1
189.6	1999	0.88	2.54	Ahstedt et al. 2016	1
189.6	2004	0.2	2.1	Ahstedt et al. 2016	1
189.6	2004	0.32	0.5	Ostby 2005	39
192.3	2007	0.06		Jones et al. 2014	2
192.4	1989-1990	0.1	0.45	Hubbs et al. 1991	37
199.1	2009	0.36		Ostby et al. 2014	40
199.1	2010	0.2		Hua 2020	42
199.1	2010	0.55		Hua 2020	42
206.9	1979	0.25	2	Ahlstedt 1991	41
206.9	2004	0.13	0.02	Ostby 2005	39
211	2012	0.03	0.04	Ostby and Beaty 2012	43
211.1	1974	0.36	2.7	Dennis 1984	40
211.1	1988	0.1	0.2	Ahstedt et al. 2016	1
211.1	1999	0.6		Ahstedt et al. 2016	1
211.1	2004	0.2	0.2	Ahstedt et al. 2016	1
211.1	2009	0.08	0.04	Ostby et al. 2014	40
213.1	1989-1990	0.05		Hubbs et al. 1991	37
213.2	2006	0.031		Jones et al. 2014	2
213.2	2006	0.04		Jones et al. 2014	2
213.2	2006	0.108	0.01	Jones et al. 2014	2
219.2	1979	0.16	0.64	Ahlstedt 1991	41
223.6	2005	0.002	0.06	Jones et al. 2014	2
226.3	1979		0.8	Ahstedt et al. 2016	1
226.3	2009	0.06		Ostby et al. 2014	40
226.7	1989-1990	0.1	0.1	Hubbs et al. 1991	37
235.1	1979		0.1	Ahlstedt 1991	37
