# Supplementary material for: Long-term monitoring of two endangered freshwater mussels (Bivalvia: Unionidae) reveals how demographic vital rates are influenced by species life history traits
Source: PLoS One. 2021 Aug 27;16(8):e0256279. doi: 10.1371/journal.pone.0256279 (PMC8396791; doi:10.1371/journal.pone.0256279)
Supplement: Attachment — Submitted filename: Response to Reviewers.docx [file pone.0256279.s012.docx]

**Response to Reviewers:**

Comment (1) on Density Dependence:

The method for detecting density dependence in the manuscript is not acceptable (lines 433-).

Please read: Freckleton, R. P., A. R. Watkinson, R. E. Green, and W. J. Sutherland. 2006. Census error and the detection of density dependence. Journal of Animal Ecology 75:837-851

The issue is that the linear regression includes the same variable (with potentially large sampling error) as a response and explanatory variables. You wrote “This relationship was strongest when population density at a site was low, as growth rate tended to increase much more rapidly in the subsequent time interval at each site” (lines 436-438). This is a strong indication that the association is spurious. The only way you can detect density dependence from the type of data you have is to use a state-space method.

Please read: Lebreton, J.-D., and O. Gimenez. 2013. Detecting and estimating density dependence in wildlife populations. The Journal of Wildlife Management 77:12-23.

Authors Response: In review of the two above articles recommended by the editor, we agree that the method used in our original draft submission for density dependence is outdated and may lead to spurious results. Hence, we have removed the density dependence methods, results, figures and discussion from the manuscript. We plan to gather more sampling data and use appropriate methods (state space approach) to test for the potential of density dependence in our study species in a future analysis.

Comment 2 on Separation of Process and Sampling Error (Line 173- ):

“Sampling error variance was determined from the variance of the 11 annual abundance estimates. …” This part does not make any sense to me. You only had 11 samples per cite. If you estimate the variance from 11 samples, it should be the total variance. I am speculating it is something to do with including or excluding spatial variations (among cite variations). It may also be something to do with not understanding the symbol that looks like curly “F” (line 172); it is not defined anywhere. A standard approach to separate sampling error and process error is to use a state-space method. There are many papers on this topic, but I suggest you read the following paper: Holmes, E. E., E. J. Ward, and K. Wills. 2012. MARSS: Multivariate autoregressive state-space models for analyzing time-series data. R Journal 4:11-19.The paper describes a multivariate case, but it is easy to collapse down to a single variable model. The time series do not look stationary. Non-stationarity might inflate process error unless you include other processes (like density dependence or other covariates).

Authors Response: The way we calculated Sampling Variance was by calculating the average variance per site each of the 11 annual abundance sample variances. The total variance per site was the variance of each of the 11 point estimates, themselves. The Temporal variance was, therefore, calculated by subtracting sampling variance from total. The standard deviations would be the sqrt of those variances and the coefficient of variation would be the standard deviation divided by the mean. We calculated the sampling variance and temporal process variation per site and by combining the data from all three sites. Our approach and results for the variance calculations are available in Supplementary Information File S3. We also ran our data through the Multivariate Autoregressive State-Space Model in the MARSS package in program R. The results were essentially the same as our current analysis and did not tell us anything different.

Estimation of Instantaneous Mortality (Line 195-):

Do you use dynamic data or static data? In other words, do you use N_0 when N_t was recruited (dynamics) or N_0 of the same year N_t was estimated (static)? In reality, the cohort that was recruited 2004 (defined as age 0) will appear as age 1 in 2005, age 2 in 2006, etc. It is not clear what is meant by “the number in a year class at time t” (line 199). Which year class? If you took the mean of all densities of the same age, I suggest you separate them. Overall, the analysis is not sufficiently described. Note there are many ways to conduct the analysis, and each has associated assumptions. It is important to clearly describe the method and justify it (this is true with all of the methods you use).

Authors Response: For both species, we used the estimated ages ̶ as determined by a species specific von Bertalanffy equations in Jones et al. (2011) ̶ of all individuals per site and per year (2004-2014) to obtain the frequency of each age class in the river. To estimate mortality, a simple linear regression was conducted on the total age-class frequency data. We have modified our methods to read as: “where $\hat{N}$*_t_* is the total number (i.e., frequency) in an age class at time *t* obtained from all three sites and 11 census (see Figure 4), and similarly $\hat{N}$_0_ is the original number in an age class, and *Z* is the instantaneous rate of mortality [21].” This method generally is a standard approach in fisheries to estimate mortality from age-class frequency data. The estimate of Z that would be obtained from separating the frequency values per site would be the same, and further, we are not trying to estimate site specific mortality rates, our sample sizes are insufficient to do this, at least for *Epioblasma brevidens* it is, rather we are estimating a mortality rate for each species throughout the study area.

Minor Comments:

Line 198: Remove parenthesis around “t”. It looks like Z is a function of t. It should be Z times t. Done, parentheses have been removed.

Line 170: clearly define the curly “F”

Line 188: define lambda_G in words later. Ok, we clarified this section of the methods so λ_G_ is now clearly defined.

Line 189: lambda is the finite annual population growth rate, and r is the instantaneous annual population growth rate. Both are called population growth rates and have the unit of per year. The difference is one is a finite rate, and the other is an instantaneous rate. Ok, we clarified this section of the methods so each growth rate is clearly defined.

Line 191: why r has a bar, and mu has hat? Remove both bar and hat. You can delete “or mu”. It does not matter what symbols others use as long as you define your parameter clearly. Ok, we removed the bar and hat from the parameters.

Line 192: insert r bar after “the arithmetic mean”. Ok, done.

Line 200: “analogous”. It is a simple linear regression (not analogous). You probably do not need much explanation about the catch-curve analysis. It is a standard method in fisheries. For ecologists, you can just mention it is analogous to life-table analysis. Ok, done, we clarified both points suggested.

Line 201: “A” is the annual finite mortality rate, and “Z” is the annual instantaneous mortality rate. Both are per-capita rates so that they have the unit of per year. Ok, these rates were defined as suggested.

Line 201: Remove round brackets around “x” in the equation. It is interpreted as b is a function of x as it is written now. Ok, done.

Line 206: “constant recruitment”. My approach would be to make the intercept a random effect to vary based on the year of “recruitment” using a Generalized Mixed Effect Model. Then, you do not have to assume constant recruitment although your sample size may not allow you to estimate. It is worth trying. Ok, from a statistical modeling perspective, the Generalized Mixed Effect Model might be a better model choice than SLR but the assumption of constant recruitment is still a more general problem in the estimation of recruitment. Whether a GLMM or SLR is used, recruitment will vary from year to year in most natural populations, and in some cases, this variable recruitment will greatly influence the slope of the regression, and hence the estimate of mortality. The advantage of our study design, is that 11 census years are used to estimate the mean age-class frequencies, hopefully improving the reliability of the mortality estimate. Further, whether GLMM or SLR is used, the slope estimate will be very similar, so either approach is going to yield a similar estimate of mortality. If you would prefer a GLMM we can do that, but it will require us to redo and update the methods and results and some of the figures. For now, we are keeping the SLR until you advise us on your preference.

Line 207: Constant survival + constant natural mortality. Does it imply that there is non-natural mortality? If they only experience natural mortality. If natural mortality is constant, the survival rate must be constant. If there is a way natural mortality is constant but survival can vary (or vice versa), you should explain it. Otherwise, you should eliminate assumption 3. Yes, your point is well taken, this is a redundant assumption and we have removed assumption 3. Thanks for pointing this out.

Reviewers' comments:

Reviewer's Responses to Questions

**Comments to the Author**

1. Is the manuscript technically sound, and do the data support the conclusions?

The manuscript must describe a technically sound piece of scientific research with data that supports the conclusions. Experiments must have been conducted rigorously, with appropriate controls, replication, and sample sizes. The conclusions must be drawn appropriately based on the data presented.

Reviewer #1: Yes

2. Has the statistical analysis been performed appropriately and rigorously?

Reviewer #1: Yes

3. Have the authors made all data underlying the findings in their manuscript fully available?

The [PLOS Data policy](http://www.plosone.org/static/policies.action#sharing) requires authors to make all data underlying the findings described in their manuscript fully available without restriction, with rare exception (please refer to the Data Availability Statement in the manuscript PDF file). The data should be provided as part of the manuscript or its supporting information, or deposited to a public repository. For example, in addition to summary statistics, the data points behind means, medians and variance measures should be available. If there are restrictions on publicly sharing data—e.g. participant privacy or use of data from a third party—those must be specified.

Reviewer #1: Yes

4. Is the manuscript presented in an intelligible fashion and written in standard English?

PLOS ONE does not copyedit accepted manuscripts, so the language in submitted articles must be clear, correct, and unambiguous. Any typographical or grammatical errors should be corrected at revision, so please note any specific errors here.

Reviewer #1: Yes

5. Review Comments to the Author

Please use the space provided to explain your answers to the questions above. You may also include additional comments for the author, including concerns about dual publication, research ethics, or publication ethics. (Please upload your review as an attachment if it exceeds 20,000 characters)

Reviewer #1: General Comments.
This is a nice paper that presents demographic data for two rare mussel species using conventional mussel survey data. Overall the paper is well written, though tends to drag on in the discussion, and the inferences made by the authors seem plausible based on their findings. I think the paper will be useful for practitioners and therefore should be published. My only real concern is whether issues with sampling from year-to-year (i.e., differences in number of quadrats and no accounting for incomplete detection) is driving some of their results. I don’t think this is a fatal flaw, just something that needs to be discussed. Below are my specific comments and I’m happy to review this paper again, if needed.

Specific comments.

Line 145 to 152 – Unclear how the authors determined the number of quadrats sampled. The authors mention % of area but do not provide % of area per site. The authors then state number of quadrats sampled per site varied from year-to-year and so at least 80 quadrats were excavated at each site to increase precision of estimated densities. However, it’s unclear how they determined this. It seems to me that since data derived from these sampling efforts is being used to make inferences about population condition the authors should then spend some time clearly detailing how mussels were sampled, which should include some information justifying sample sizes so that readers can determine for themselves whether changes in population condition are due to natural processes or sample bias.

We made some changes to the Methods better explain the origin of the dataset in the text and in response to the specific critiques regarding dual publication. Our dataset was collected and compiled by a network of scientists and managers that recognized annual quantitative data would be necessary to understand populations of these rare species. We were initially constrained by an ethical concern that oversampling might have deleterious effects on mussels. The sites we visited are among the best remaining in the world and some studies have suggested that sampling might have adverse physiological effects.

The network of scientists and managers used funding from various sources and help from many institutions. Sampling was initiated by Jess Jones in 2004 and at that time, its use was specific to project goals; it was intended to provide abundance estimates for a discrete time period (2004-2008). Several partners were encouraged by the breadth of information gleamed from that sampling approach and helped continue sampling over the next 7 years. As work proceeded, we gained a better understanding of variation increased sampling to minimize bias and improve detection of rare species. The variation in sampling was a function of practical and ethical constraints and also an example of learning and adapting. More recent data should be considered less biased but not so different that comparison to earlier data is invalid.

At no point in previous publications was the entire dataset from 2004-2014 examined. Our study specifically focused on these two endangered species with the specific purpose of deriving key demographic parameter estimates. Our analyses are new and far more through than previous attempts that examined the mussel assemblage (30 plus species) as a whole, which has been published elsewhere. Earlier analyses focusing on these species only used data from 2004-2008 and had different objectives and fare different methods. Also novel to this analysis was the examination of flow data.

Line 164: The authors state that logarithmic transformation of the sample estimate was used to calculate 95% confidence limits if data were non-normally distributed. I find this statement confusing given that in the previous sentence the authors stated that each census sample was evaluated for normality. My understanding is that log transformation is often used to deal with skewed data, which if uncorrected can yield invalid statistical results using parametric statistical tests. However, log transformations only work if the original data follow a log-normal distribution. So, my question is what does 95% CI have to do with addressing non-normally distributed data and did the transformation fix their issue?

In the Methods we modified this sentence to read as “Because all site density was not normally distributed, it was right-skewed due to the quadrat sample data being inflated with zeroes and ones, a logarithmic transformation of the sample estimate was used to calculate 95% confidence limits (CL) if data were non-normally distributed”. So, yes, our data is distributed log-normally, being right skewed, and therefore we used a non-parametric approach to calculate the confidence limits. The data itself was not log-normally transformed.

Line 182: It would be helpful if the authors provided more details on the age estimates. Simply stating ages of live mussels were estimated using von Bertallanfy growth equations doesn’t tell you anything beyond how age was analytically determined. That is, did the author’s thin-section shell to determine age-length relationships or did they use external annuli or some combination of both? Ok, we fixed this by adding to this sentence so it now reads as “Ages of live mussels were estimated using von Bertallanfy growth equations [19] specific to females and males of each species; the equations and associated shell growth parameters were derived from shell thin-sections to determine age [4].

Line 206: I wonder if just truncating age cohorts really addresses violations of these assumptions? For example, do we really know whether recruitment is constant from year-to-year? Or how just omitting size classes less than 1 year old minimizes violation of assumptions 2 and 4? Presumably mortality would be higher in younger cohorts but where you draw the line is anyone’s guess. Same goes for natural mortality. Do the authors have any sense of how violations of these assumptions influence their estimates? It seems to me a more productive way to handle this is to talk about what they did (i.e., omitting smaller size individuals) but then elaborate on how violations of these assumptions affect their inferences. As GEP Box once said, “All models are wrong, but some are useful.” In that spirit, the authors should help clarify the uncertainty about the true parameter values – in this case mortality. It is well known in the fisheries literature that these assumptions are regularly violated, especially the constant recruitment assumption, which obviously is unrealistic. We have conducted the catch-curve analyses both ways, by removing the young individuals that do not ”recruit” well to the sampling gear because they are too small and harder for surveyor’s to detect as the larger adults, and with the larger adults. So readers have the two estimates to compare, which is a useful approach. But most importantly, our study incorporates 11 censuses of age-size-class frequency data, which allows us to take the mean over a very significant time period to estimate mortality. This approach alleviates many of the concerns of just conducting a catch-curve mortality estimate just based off of one year of data. Hence, our study is robust to many of the assumptions.

Line 229: Just caught this but the authors switch from using scientific to common names. My recommendation is to use scientific names throughout. Ok, this has been changed as suggested.

Line 251: The authors may want to consider using IHA (Indicators of Hydrological alteration) to calculate annual flow statistics that may be helpful in explaining year-to-year variation in growth, longevity and survivorship. Rypel et al. (2009) takes this approach to explain differences in mussel growth between regulated vs. unregulated rivers.

We used this opportunity to examine flow data using IHA software. Its use was insightful and helped us to correct some issues with flow statistics, including an incorrect assignment of a low-flow phenomena to an interval and an imprecise calculation of extremes. At the same time, it provided many more statistics that had to be examined using a more intensive statistical approach; that being an examination of covariation and comparison of competing hypotheses. IHA also produced a means to examine whether the study period was different or special. Ultimately, IHA application did not alter the conclusion that flow was associated with population growth, and that that variation was overwhelmingly related to juvenile recruitment. IHA also highlighted that study period had a unique period of low flow and paucity of flood events. Beyond that, many statistics and analysis within IHA were intended to compare regulated streams to unaltered system, so those statistics were not used in deference to more intuitive statistics we though were related to life history.

Results – This goes back to my question regarding details on sampling. I wonder how much of the lack of significance reported throughout or even the trends noted by the authors is due to sampling bias. It seems to me that varying level of effort (i.e. different number of quadrats sampled from year-to-year) and no accounting for detection could be driving things. I think the authors need to address this somewhere, maybe in the methods section and then provide a paragraph in the discussion that talks about limitations of their findings and future opportunities to build off this research and validate their findings.

Line 542: This is a great paragraph but I wonder how much of this “sampling variation” is due to differences in effort between years and not accounting for incomplete detection.

A few things here to consider when we are attempting to detect real population trends versus noise. First, quadrats have a high probability of detecting mussels within units, >0.99 for adults, about 0.7 to 0.9 for juveniles depending on their size. Second, these mussels are present at moderate densities for mussels, 0.25 m^-2^ or even at much greater densities. Thus, increasing our sample size from 60 to 80 to 100 units does not really change our ability to detect the mussels. It would make a big difference if the quadrat sample units had poor detection. So, the variation among years we observed in study is real, even though confidence intervals are wider than we would like. We would probably have to multiply our quadrat sample size by a factor of 10 to get really narrow confidence intervals to define site densities with absolute precision. The problem with such a high quadrat sample size is that we would do significant damage to the habitat and mussels resources by doing such an intensive sampling design.

There is some evidence that sampling affects growth and perhaps even survival of some species. The handing of mussels, while typically not lethal, can stress the mussels and might even be considered harassment. Many of the species we study are endangered and extremely rare, so we cannot put ethics aside in pursuit of mathematical certainty. The data used in this analysis was collected while simultaneously sampling many rare and protected species. As we move forward with management, we either have this information or none at all. Continuing to sample over time will allow us to better distinguish between noise and signal.        

Line 625: Bonanza, really? Delete “a bonanza in” Ok, we have deleted this phrasing so it now reads as “with a large increase in positive population growth”

Line 642: The age structure, demographic vital rates and life history section is too long, and it needs to be revised. I’ve read it several times now and I’m still not 100% sure what exactly they are trying to say. My suggestion is for them to introduce life history theory in the beginning and then present their findings so it’s clear which strategy their focal species belong to. While doing this they could then discuss how these traits may explain mussel-environmental relationships observed during their study. They should also talk about how these traits could be used to inform future conservation activities for both species. Winemiller (2005) does a good job doing this but uses fish instead of mussels.

We have restructured the discussion and reframed the study objectives to help the reader. We have not reworked as specified but found a different way to narrow the scope and focus the reader’s attention to key conclusion and applications of this analysis.
