# Supplementary material for: Long-term monitoring of two endangered freshwater mussels (Bivalvia: Unionidae) reveals how demographic vital rates are influenced by species life history traits
Source: PLoS One. 2021 Aug 27;16(8):e0256279. doi: 10.1371/journal.pone.0256279 (PMC8396791; doi:10.1371/journal.pone.0256279)
Supplement: Attachment — Submitted filename: Response to Reviewers.docx [file pone.0256279.s013.docx]

PONE-D-20-36478R1
Long-term Monitoring of Two Endangered Freshwater Mussels (Bivalvia: Unionidae) Reveals How Demographic Vital Rates Are Influenced By Life History Traits of Each Species
PLOS ONE

Journal Requirements:

Please review your reference list to ensure that it is complete and correct. If you have cited papers that have been retracted, please include the rationale for doing so in the manuscript text, or remove these references and replace them with relevant current references. Any changes to the reference list should be mentioned in the rebuttal letter that accompanies your revised manuscript. If you need to cite a retracted article, indicate the article’s retracted status in the References list and also include a citation and full reference for the retraction notice.

**RESPONSE**: The references have been checked and all citations in the text are included in the reference list, no additional citations have been added and none have been removed from the previous version.

Additional Editor Comments (if provided):

Some brief discussions on the representativeness of the samples may be useful for the readers. This relates to the first comment provided by the reviewer on the previous version.

**RESPONSE**: Lines 163-167 have been added in the Methods describing the representativeness of the samples.

Line 179: I am still confused with the log transformation. You wrote that the data include zeros. How did you handle the natural log of 0? Some clarification will be helpful.

**RESPONSE**: Since our data was right skewed, we used the method of Strayer and Smith 2003 (Citation #16) for calculating the 95% confidence limits (CL’s). The method uses a logarithmic transformation of the site mean density (D) and a delta-method approximation of the variance to estimate the CL’s. So quadrat samples with no mussels, i.e., 0 values were not transformed, only the site mean and variance of D were transformed to obtain the CL’s. The method has been applied widely to quadrat sampling of freshwater mussel communities to estimate CL’s. We have updated this part of the sentence on line 179 to make it more specific, inserted the formula we used and added the appropriate citation in case readers are interested in this method for calculating CL‘s when the underlying quadrat data are right skewed.

Line 207: What distribution was used (Gaussian?) for 95% CI?

**RESPONSE:** We assumed a normal (Gaussian) distribution to compute the variance and standard error for each species’ respective population growth rate. However, we did not compute the 95% CI’s for this parameter.

Line 222: A major assumption missing is that all age/size classes have the same catchability, which may be violated with your data (see the comment below).

Line 463: Looking at Figure 8, I suspect ages 0-3 probably have lower catchability. If so, those age classes should be eliminated from the catch-curve analysis. Then, Figure 8 should just include the line fitted to data without ages 0-3. If not, further discussion is probably needed.

**RESPONSE:** It is important that Ages 1-3 remain in our analyses, as shown in Figure 8 and as reported in the Results. I’ve updated the figure legend to make it clear that estimates of mortality are available with and without Ages 1-3 included in the analyses. Our analyses do not include Age 0 individuals but our original graphs do extend to the y-axis, giving the impression that Age-0 individuals were included in the analysis. So we have remade the graph to make it clear that Age 0 individuals were not included in the analysis. Generally, we believe that Ages 1-3 have equal catchability relative to the older larger mussels but perhaps lower mortality but that as individuals mature and age their mortality increases, so having mortality rate estimates with and without Ages 1-3 is important for readers to see how the mortality rate will likely increase in the older adult age classes.

Line 569: Correlation analysis is fine, but it is better to use GLM or GAM with careful assessments of distribution, residuals, and fit (although I do not insist on this).

**RESPONSE**: We used a two-step process for developing competing linear regressions (a form of GLM).  Correlation analysis was presented as a screening analysis to inform ultimate linear regression models (our hypotheses).  We had many possible independent variables and few independent replicates of dependent variables.  Many independent variables were correlated.  We used correlation analysis to better inform which of many competing independent variables to include in final model runs (informed hypotheses).  To a more limited degree, we did this for dependent variables.  We chose only one or two of correlating independent variables that we felt were possibly biologically significant for final models.  Final linear regression models were GLMs. GLMs are generally robust to problems with distribution and were used because were looking for linear relationships.  We did not think other distributions were appropriate to explore at this time. We used AICc to compare relative support, so fit statistics, such as r2, are not appropriate. As we collect more data at these sites we hope to build and compare other models. GAM may be more appropriate at a later point when we have more than 10 independent replicates.  At this point there is no advantage to use GAM over information theory approach to compare competing linear regression models.     

Line 672: CMR sampling has been done with freshwater mussels previously by others (I believe). It may be helpful for the readers if the pros and cons of CMR and the catch curve analysis are briefly discussed. It is also useful to mention that the state-space method was applied but it is not shown because most of the variation was from sampling errors.

**RESPONSE**: CMR sampling is increasingly popular and authors have been using this approach at other sites and with other mussel species. Two of the coauthors were also coauthors of Carey et al. 2019--a study that compared CMR techniques to quadrat monitoring. We have decided not to go into a discussion of the pros and cons of CMR because it would be lengthy and deserves a standalone review that would expand on the work of Carey et al. 2019.  Moreover, CMR would be no more effective due to relative rarity of E. brevidens, which is distributed at low levels across large sties.  Seconding, applying CMR at these sites would mean we could not collect data on the dozens of other species we monitor at these sites.  Finally, CMR approaches at these sites may put too much pressure on sensitive areas where we limit our impact.  Previous CMR studies on the Clinch River required 5 sampling occasions each year for 2 years (Carey et al. 2019) and only produced limited information about those 2 years.  As even short-lived mussels live 5 yrs or more, understanding population dynamics over longer time spans is critical.

[Note: HTML markup is below. Please do not edit.]

[NOTE: If reviewer comments were submitted as an attachment file, they will be attached to this email and accessible via the submission site. Please log into your account, locate the manuscript record, and check for the action link "View Attachments". If this link does not appear, there are no attachment files.]

While revising your submission, please upload your figure files to the Preflight Analysis and Conversion Engine (PACE) digital diagnostic tool, <https://pacev2.apexcovantage.com/>. PACE helps ensure that figures meet PLOS requirements. To use PACE, you must first register as a user. Registration is free. Then, login and navigate to the UPLOAD tab, where you will find detailed instructions on how to use the tool. If you encounter any issues or have any questions when using PACE, please email PLOS at [figures@plos.org](mailto:figures@plos.org). Please note that Supporting Information files do not need this step.
